# Regulation of Gastrointestinal Immunity by Metabolites

**DOI:** 10.3390/nu13010167

**Published:** 2021-01-07

**Authors:** Bon-Hee Gu, Myunghoo Kim, Cheol-Heui Yun

**Affiliations:** 1 Life and Industry Convergence Research Institute, Pusan National University, Miryang 50463, Korea; g.bonhee@gmail.com; 2 Department of Animal Science, College of Natural Resources & Life Science, Pusan National University, Miryang 50463, Korea; 3 Department of Agricultural Biotechnology and Research Institute of Agriculture and Life Sciences, Seoul National University, Seoul 08826, Korea; 4 Center for Food and Bioconvergence, Seoul National University, Seoul 08826, Korea; 5 Institute of Green-Bio Science and Technology, Seoul National University, Pyeongchang-gun, Gangwon-do 25354, Korea

**Keywords:** bile acids, dietary metabolites, intestinal homeostasis, metabolism, microbial metabolites, microbiota, short-chain fatty acids, tryptophan

## Abstract

The gastrointestinal tract contains multiple types of immune cells that maintain the balance between tolerance and activation at the first line of host defense facing non-self antigens, including dietary antigens, commensal bacteria, and sometimes unexpected pathogens. The maintenance of homeostasis at the gastrointestinal tract requires stringent regulation of immune responses against various environmental conditions. Dietary components can be converted into gut metabolites with unique functional activities through host as well as microbial enzymatic activities. Accumulating evidence demonstrates that gastrointestinal metabolites have significant impacts on the regulation of intestinal immunity and are further integrated into the immune response of distal mucosal tissue. Metabolites, especially those derived from the microbiota, regulate immune cell functions in various ways, including the recognition and activation of cell surface receptors, the control of gene expression by epigenetic regulation, and the integration of cellular metabolism. These mucosal immune regulations are key to understanding the mechanisms underlying the development of gastrointestinal disorders. Here, we review recent advancements in our understanding of the role of gut metabolites in the regulation of gastrointestinal immunity, highlighting the cellular and molecular regulatory mechanisms by macronutrient-derived metabolites.

## 1. Introduction

The phrase “You are what you eat” emphasizes the importance of diet on human health. The diet contains various components including proteins, lipids, carbohydrates, and nucleic acid building blocks, which supply an energy through their metabolism. Metabolic reactions, regulated by series of enzymatic reactions, convert dietary nutrients into diverse metabolites, and most of which responses occur in the gastrointestinal tract. Enzymes secreted from host intestinal tissues digest dietary components to produce various metabolites, which are mostly absorbed at the small intestine. In the gastrointestinal tract, unlike other tissues, a huge portion of the gut microbiota colonizes and plays diverse functions including the integration of gut metabolism. Undigested nutrients or gut metabolites are further processed through gut microbial fermentation, which produces new types of microbial metabolites by microbial enzyme activities. As the spectrum of gut microbial enzymes is different from those of the host enzyme, one could expect more diverse gut metabolite profiles in the gastrointestinal tract. On the other hand, the composition of the gut microbiota is easily influenced by diet, and different microbiota communities produce distinct gut microbial metabolites. Thus, type of diet is considered to be a key regulatory factor for the gut metabolic profile through manipulation of the microbiota community. Collectively, dietary components are converted into diverse dietary and microbial metabolites by complex enzymatic reactions and microbial fermentation, respectively.

The maintenance of immune homeostasis in mucosal tissues is quite challenging as the mucosal barrier is vulnerable to damage by various extrinsic factors including diet, microbiota, and toxins. To maintain immune homeostasis, under steady state conditions, inflammatory responses are restrained through mechanisms that promote the regulation of immune cells and fortifying barrier functions in mucosal tissues. Disruption of such regulation contributes to inflammation and, if severe, autoimmune diseases. It is generally accepted that the occurrence and severity of these inflammatory diseases are regulated by various extrinsic factors including the diet and microbiota. It is worthwhile noting that the most prominent ways in which the diet and microbiota affect mucosal immunity are through the production of metabolites. The intestinal tissue is the major target for gut-metabolite-derived signals. Dietary and microbial metabolites directly regulate epithelial cell and intestinal immune cell function, which play significant roles in the maintenance of immune homeostasis in the gut. Accumulating reports further suggest that metabolites largely influence the development of gut inflammatory diseases such as inflammatory bowel diseases and colorectal cancer through diverse immune regulatory mechanisms [1,2]. An integrative approach, including immunology, nutrition, and microbiology, is needed to better understand the link between diet and immune diseases. In this article, we review the current knowledge on the link between nutritional immunology and gut metabolites in the regulation of gastrointestinal immunity.

## 2. Intestinal Immunity

The gastrointestinal tract is a highly complex organ, which includes the intestinal epithelium layer, lamina propria, mucus, microorganisms, dietary materials, enteric nervous system, and local immune system. Particularly, the organized immune structures of gut-associated lymphoid tissue (GALT) and the draining of lymph nodes in the intestine act as principal sites for priming adaptive immune responses [3]. The GALT contains microfold (M) cells and Peyer’s patches (PPs) and comprises the subepithelial lymphoid aggregates that lie in the mucosa and submucosa. As M cells normally have high levels of phagocytosis and transcytosis activity, they are specialized for the uptake of intact antigens that are normally unable to enter the lamina propria from the lumen, where dendritic cells (DCs) process and present antigens to T cells [4]. This provides an important route for intestinal immune cells to recognize luminal antigens. PPs lie in the small intestine, which contains numerous B cell lymphoid follicles and the T cell area [5]. PPs are known to be a major source of small intestinal IgA, which is one of the key components of the mucosal protective immune system. GALT also includes smaller lymphoid aggregates such as cryptopatches and isolated lymphoid follicles. These lymphoid structures also contain adaptive immune cells, particularly B cells. The composition of immune cells and even the structural formation of these smaller lymphoid aggregates change due to ageing as well as with the colonization and diversity of the gut microbiota [6]. Mesenteric lymph nodes (MLNs) are the largest lymphoid tissue in the body, where constant exposure of immune-stimulatory antigens to the gastrointestinal area and interactive responses between innate and adaptive immune cells are observed. It is worthwhile noting that antigens from each anatomical location throughout the gastrointestinal tract drain into distinct lymph nodes [7]. This suggests that dynamic interactive responses between innate and adaptive immune cells occur differently at various lymphoid tissue sites.

The intestine contains the largest number of immune cells compared with any other tissues in the body [3]. The epithelium and lamina propria are the effector sites of the intestinal immune system and contain different immune cell populations. While a relatively smaller number of immune cells exist within the intestinal epithelium than those of the lamina propria, the intestinal epithelium acts as a central coordinator of mucosal immunity. Accumulating reports suggest that dietary and microbial metabolites control epithelial functions [8,9,10]. Intestinal epithelial cells (IECs) are composed of several subtypes, including enterocytes, goblet cells, paneth cells, tuft cells, enteroendocrine cells, and M cells. They are differently distributed in small and large intestine and play distinct roles in the intestinal biology [11]. The major immunological function of IECs is to provide a barrier between the inner and external (luminar) sides. The intestinal epithelial layer provides a physical and chemical barrier between luminal immunogenic agents and the host immune system to prevent undesired activation of immune cells in the intestinal tissue. Therefore, epithelial barrier integrity is a critical factor in the maintenance of intestinal tissue homeostasis, a process that is influenced by the gut microbiota and metabolites [10,12]. IECs also actively participate in the orchestration of intestinal immune responses through the recognition of luminal antigens and the production of immune modulatory molecules. IECs produce a variety of functional molecules, including cytokines, chemokines, and anti-microbial peptides, that act as key regulators of intestinal immune responses. IECs express multiple types of pattern recognition receptors (PRRs) including Toll-like receptors (TLRs), C-type lectin receptors, retinoic acid-inducible gene (RIG)-I-like receptors (RLRs), nucleotide-binding oligomerization domain (NOD)-like receptors (NLRs), and absent in melanoma 2 (AIM2)-like receptors (ALRs) [13,14]. Like other innate immune cells, PRRs on IECs recognize pathogen-associated molecular patterns (PAMPs) or danger-associated molecular patterns (DAMPs). This epithelial recognition provides a critical initial step for crosstalk between luminal microbes and intestinal immunity. In response to PAMPs and DAMPs, IECs produce various immune modulatory cytokines and host defense peptides such as antimicrobial peptides (AMPs), which mediate gut barrier function. In addition, IEC-derived cytokines shape intestinal immune cell responses in the lamina propria. For example, IECs produce alarmin cytokine interleukin (IL)-25, IL-33, and thymic stromal lymphopoietin (TSLP) as endogenous damage signals that play significant roles in the regulation of immune cell activation. These cytokines are known to induce the activation of type 2 innate lymphoid cells (ILC2) to secrete IL-5 and IL-13 in the context of type 2 immunity [15,16,17]. IECs also receive internal signals such as cytokines that are recognized by their receptors to respond and control intestinal immune responses. In response to IL-22, paneth cells secrete AMPs such as lysozyme and defensins, which are critical for the anatomical compartmentalization and maintenance of symbiotic microbes [18]. IECs also secrete IL-18, linked to inflammasome activation after the engagement of microbial and host-derived molecules through both direct and indirect methods [18]. As IL-18 is a critical cytokine for the induction of AMPs, impaired inflammasome activation in IECs induces the dysbiosis of gut microbiota resulting in inflammatory disease [19,20]. Collectively, IECs are central mediators that orchestrate intestinal immune responses in the middle of external stimuli and intestinal immune cells.

Mononuclear phagocytes, consisting macrophages and DCs, share the expression of certain markers such as CD11b, CD11c, and major histocompatibility complex (MHC)II [21]. They take up antigens and present antigenic peptides to adaptive immune cells with pro-inflammatory cytokine production. The majority of intestinal macrophages are found in healthy intestinal lamina propria [21]. They play critical roles in controlling intestinal homeostasis through the clearance of pathogens and cellular debris. Most tissue-resident macrophages in the gut lamina propria express C-X_3_-C motif chemokine receptor 1 (CX_3_CR1), which regulates the homeostasis of intestinal macrophages and bacterial translocation. CX3CR1^+^ macrophages extend the transepithelial dendrites between IECs to capture food and bacterial antigens in the lumen and finally destroy the captured antigens with their high levels of phagocytic and bactericidal activity [22]. In addition, CX3CR1^+^ macrophages, referred to as anti-inflammatory (M2) macrophages, maintain intestinal homeostasis via IL-10 production, which facilitates the expansion and maintenance of Foxp3^+^ regulatory T cells (Tregs) [22]. Intestinal DCs in the lamina propria can be subdivided by their CD11b and CD103 expression into four subsets that are differently distributed throughout the intestinal tissues. For example, CD11b^+^CD103^+^ DCs are the major subset in the small intestine, while CD11b^−^CD103^+^ DCs are the major DC subsets in the colon. It has been reported that CD11b^+^CD103^+^ DCs correlate with Th17 responses [21]. Although the molecular mechanism for balancing T cell homeostasis using specific types of DCs is yet to be further defined, Th17-skewing cytokine IL-6 from CD11b^+^CD103^+^ DCs could explain such regulation. In the lamina propria, plasmacytoid DCs (pDCs) are also present but in smaller numbers than conventional DCs. The pDCs appear to be recruited to the colonic mucosa in response to inflammation or microbial infection [23,24]. It has been reported that pDCs sense the microbiota induce naïve T cells into IL-10, producing CD4^+^ T cells [25]. Intestinal DCs play a role in the regulation of intestinal immunity through the manipulation of intestinal T cell responses. It is noteworthy that regulatory functions and specific subset distributions of DCs and macrophages can be influenced by dietary and microbial metabolites.

The intestine contains both innate and adaptive lymphocytes. Innate lymphocytes including innate lymphoid cells (ILCs), eosinophils, mast cells, invariant T cells, and intraepithelial lymphocytes (IELs) are distributed along the gastrointestinal tract. ILCs, most recently identified, are a type of innate lymphocytes that are primarily localized in mucosal tissues such as the intestine. ILC1, ILC2, and ILC3 resemble Th1, Th2, and Th17, respectively, in terms of their cytokine expression and the transcription factors involved [25]. Intestinal ILCs receive immune-stimulatory signals from microbes, metabolites, or dietary antigens to produce cytokines that significantly affect intestinal homeostasis and inflammation [25]. A number of eosinophils and mast cells always exist in the gastrointestinal tract and contribute to multiple phases of the immune response, including innate immunity, adaptive immunity, and tissue repair [26,27]. While eosinophils and mast cells have homeostatic functions in heathy intestinal tissue, they increase in concentration in the intestine where they work together with ILC2 to produce type 2 immunity, which is involved in disease progress and the tissue repair process [28,29]. A minor proportion of T cells that express invariant forms of the T cell receptor, such as mucosal-associated invariant T cells (MAIT cells) and invariant natural killer T cells (iNKT cells), are found in the intestinal tissue [30]. MAIT cells are only found in the human intestine and mediate innate immune functions including the production of cytokines and the execution of cytolytic activity. Interestingly, MAIT cells recognize vitamin B metabolites produced by the gut microbiota. The iNKT cell, produces IL-4 and IFN- γ in response to glycolipids from the host and bacteria [30]. Another type of innate lymphocyte, the IEL, is mainly located within IECs that have regulatory and effector activities via the induction of intestinal inflammation and epithelial homeostasis [31]. T and B lymphocytes provide a specific type of immunity to luminal antigens. T cells are located in both the intestinal epithelium and the lamina propria. In the lamina propria, distinct CD4^+^ T cell subsets, Th1, Th2, Th17, and Tregs, exist depending on the immune milieu in the intestinal tissue. Naïve T cells recognize antigens by antigen-presenting cells (APCs) in the induction sites of lymphoid tissues and then migrate into effector sites. The balance among T cell subsets is critical for the maintenance of tissue homeostasis [32,33]. Largely, the balance between pro-inflammatory T cells (Th1, Th2, Th17) and anti-inflammatory T cells (Treg) controls immune homeostasis in the intestinal area. Recent studies reported that gut metabolites change the balance of CD4^+^ T cell subsets. Short-chain fatty acids (SCFAs), which are microbial metabolites produced as a result of carbohydrate utilization, expand the Treg pool in the gut through diverse molecular mechanisms [34]. A large number of B cells are also found in the lamina propria; however, IgA expressing plasma cells are mainly located in intestinal lymphoid tissues, such as PPs and MLNs. Secretory immunoglobulin A (sIgA) plays a critical role in controlling pathogens and commensal bacteria in the lumen of the intestine. Dietary regulation of intestinal B cell responses through gut metabolites has been reported [35,36]. Collectively, innate immune cells (IECs, macrophages, DCs, ILCs, mast cells, eosinophils, MAIT cells, and iNK T cells) and adaptive immune cells (IgA^+^ B cells and T cells) work together to maintain immune homeostasis in the gut under steady state conditions or during the healing process and to fight against pathogens after the infection.

## 3. Dietary and Microbial Metabolites Produced in the Gastrointestinal Tract

Various metabolites are produced via host and microbial metabolism, mainly in the gastrointestinal tract. Gut metabolites participate in energy metabolism, cell-to-cell interactions, and host immune responses [37,38,39]. The small intestine is the most active site for nutrient digestion in the context of host metabolism. The majority of host enzymes are secreted into the lumen of the small intestine, and therefore, various metabolites, such as lactate, are produced and absorbed.

The gastrointestinal tract is a digestive organ that is rich in dietary materials and provides an ideal niche for the gut microbiota. A variety of distinct microbial habitats are found in different anatomical locations along the gastrointestinal tract. Physiological variation, defined by complex gradients of chemical, physical, nutritional, and immunological factors, is known to influence the composition of the bacterial community [40]. The gut microbiota plays a pivotal role in host health through the integration of host metabolism and immunity [41,42]. While many gut microbiota studies have focused on the regulation of host immunity, the overall metabolism potential has been under-explored. In the gastrointestinal tract, gut microbes with a metabolically active status harvest energy from dietary components including carbohydrates, proteins, and lipids through microbial fermentation [37]. The gut microbiota generates metabolites by directly converting components or modifying host-derived metabolites (e.g., bile acids). Additionally, the gut microbiota synthesizes metabolites de novo that the host cannot produce (e.g., SCFAs, vitamins). The microbial composition and diversity differ along the gastrointestinal tract because of different microenvironmental conditions (e.g., pH, retention time, the influx of digestive enzymes and bile acids). In humans, the microbial density of the small intestine ranges from about 1 × 10^3^ CFU/g of luminal content in the duodenum to 1 × 10^8^ CFU/g in the ileum [43]. Firmicutes (*Streptococcaceae*) and Proteobacteria (*Enterobacteriaceae*) are the dominant phyla in the small intestine. In comparison, the human colon harbors about 1 × 10^11^ CFU/g of the luminal content and is dominated by Bacterioidetes (*Bacteroidaceae*, *Prevotellaceae, Rikenellaceae*) and Firmicutes (*Lachnospiraceae and Ruminococcaceae*) [43,44,45]. The variation in the physiological characteristics of these microbial communities results in the production of unique metabolites in each region of the gastrointestinal tract. Thus, the abundance and nature of metabolites are outcomes of the diversity and composition of the gut microbiota. Collectively, diverse metabolites are produced by host and microbial enzymatic reactions in the gastrointestinal tract.

### 3.1. Production of Dietary Metabolites in the Small Intestine

#### 3.1.1. Carbohydrate Metabolites

**Figure 1:**
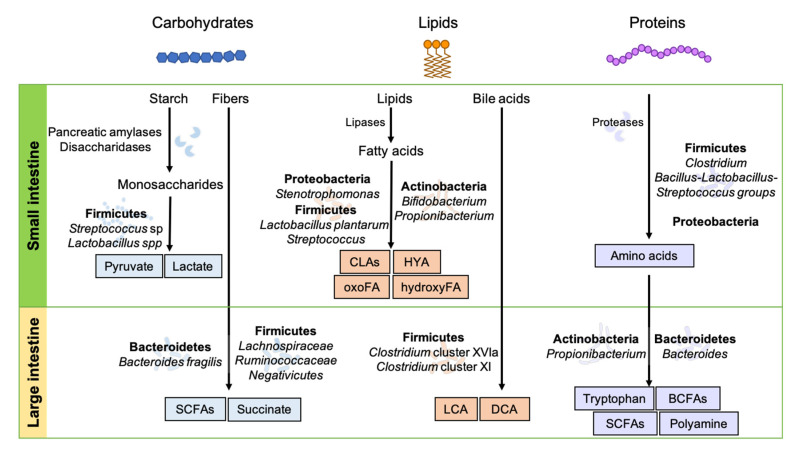
Dietary metabolism in the gastrointestinal tract. Dietary components—carbohydrates, lipids, and proteins—are digested into diverse metabolites via complex reactions of enzymes released from host tissues and microbial fermentation in the gut. BCFAs, branched chain fatty acids; CLAs, conjugated linoleic acids; DCA, deoxycholic acid; HYA, 10-hydroxy-cis-12-octadeccenoid acid; hydroxy FA, hydroxy fatty acid; LCA, lithocholic acid; oxoFA, oxo fatty acid; SCFAs, short chain fatty acids.

The small intestine is the major region for the digestion and absorption of carbohydrates that are utilized by host tissues and gut microbes (Figure 1). Digestive enzymes such as disaccharidases released from enterocytes hydrolyze carbohydrates to monosaccharides (e.g., glucose, fructose, and galactose), which undergo glycolysis to create pyruvate for energy production [46,47]. Under anaerobic conditions, pyruvate is converted into lactate by lactate dehydrogenase [48]. In paneth cells, lactate is a critical metabolite to maintain the proliferation and differentiation of small intestinal stem cells for gut homeostasis [49]. Intestinal stem cells convert lactate into pyruvate and enhance oxidative metabolism, leading to reactive oxygen species (ROS)-induced intra-cellular signaling for differentiation [49].

Although, a limited number of gut microbes exists in small intestine, some small intestinal microbes, including *Lactobacillus* spp., play significant roles in the regulation of host metabolism and immunity [50]. In addition to host cells, commensal bacteria not only aid in carbohydrate digestion through the release of lactase, which hydrolyzes lactose to glucose, but also produce carbohydrate metabolites, resulting in the maintenance of gut homeostasis [50]. In metagenomic and metatranscriptomic analyses with human ileostomy, the small intestine has been shown to be enriched with genes expressing *Streptococcus* spp., allowing phosphotransferase systems to take up monosaccharides and carry out carbohydrate metabolism, including glycolysis and pentose phosphate pathways [45], and consequently produce lactate (Figure 1).

#### 3.1.2. Lipid and Bile Acid Metabolites

Lipid digestion, known as lipolysis, and its absorption primarily occur in the duodenum and jejunum. Lipases play essential roles in the digestion, transport, and processing of dietary lipids (i.e., triacylglycerols, fats and oils) in animals and humans and act at a specific position on the glycerol backbone of the lipid substrate [51]. For instance, gastric lipases break down triglycerides into diglycerides and fatty acids, and pancreatic lipases convert emulsified lipids into fatty acids, monoglycerides, and glycerol. Phospholipases and sphingomyelinases are also seen in nature, yet they are generally treated separately from the aforementioned lipases. Fatty acids are precursors for the synthesis of several lipid metabolites. Fatty acids and their metabolites act as energy sources and membrane components. Among the four types of long-chain fatty acids (saturated, monounsaturated, polyunsaturated, and trans), polyunsaturated fatty acids (PUFAs) are required for a variety of physiological processes and are mostly considered to be essential fatty acids, because animal and human cells cannot synthesize them and thus must be supplied by the diet. Linoleic acid (ω6) and α-linolenic acid (ω3) are two major PUFAs. A metabolite of linoleic acid (ω6) is arachidonic acid, which is further metabolized into leukotrienes, lipoxins, prostaglandins, and thromboxane-prostanoid. The major metabolites of α-linolenic acid (ω3) are eicosapentaenoic acids and docosapentaenoic acids, which serve as precursors to specialized pro-resolving mediators, such as resolvins, protectins, and maresins [52]. The metabolic pathways of linoleic acid and α-linolenic acid require the same series of oxidative enzymes: cyclooxygenases, lipoxygenases, and cytochrome P450 monooxygenases. Thus, they compete among themselves for immunological outputs [53].

Lipid metabolites are generated by not only the host but also microbiota expressing lipid metabolizing enzymes in the intestinal tract. Indeed, a low microbial gene richness in the human gut is related to impaired lipid metabolism and an increased risk of developing metabolic diseases [54,55]. In animal studies, germ-free (GF) mice have greater lipid excretion and reduced lipid metabolites with impaired lipid digestion and absorption in the small intestine [56,57]. However, the impaired lipid metabolism in GF mice was recovered by transplantation of high-fat-diet-induced jejunal microbiota with increased abundance of *Clostridiaceae* and decreases in *Bifidobacteriaceae* and *Bacteroidaceae* [58]. *Lactobacillus plantarum* expresses polyunsaturated fatty acid-saturating enzymes, which are known to generate conjugated linoleic acids (CLAs), hydroxy fatty acids, and oxo fatty acids [59]. *Bifidobacterium* strains and *Propionibacterium freudenreichii* also produce CLAs through the activity of specific CLA-converting enzymes. 10-Hydroxy-cis-12-octadeccenoid acid (HYA) is produced by FAD-dependent myosin cross-reactive antigen protein expressing bacteria (i.e., *Lactobacillus*, *Bifidobacterium*, *Streptococcus*, and *Stenotrophomonas*) (Figure 1) [60,61,62].

Bile, mainly constituted by bile acids and cholesterol, is synthesized in the liver. It emulsifies and solubilizes lipids, thereby playing a critical role in fat digestion. Cholesterol and bile acids are important for the regulation of digestive function, nutrient metabolism, and immune responses. Most cholesterol is absorbed in the duodenum and proximal jejunum through a passive diffusion process. Reabsorbed cholesterol is incorporated into triglycerides and lipoproteins, forming transportable complexes called chylomicrons, which return to the liver via the enterohepatic circulation. Primary bile acids (e.g., cholic acid, chenodeoxycholic acid, hydrochloric acid) are synthesized from cholesterol in the liver and secreted as taurine or glycine-conjugated forms into the duodenum of the small intestine to aid in the emulsification of dietary lipids and fat-soluble vitamins. In the small intestine, the secreted bile acids undergo deconjugation by microbes to produce bile salt hydrolases. Most bile acids (≥95%) are reabsorbed in the terminal ileum and reused through the enterohepatic circulation [63].

#### 3.1.3. Protein Metabolites

A substantial portion of protein metabolism occurs in the small intestine [45]. Digestion of dietary proteins starts in the stomach with pepsin and hydrochloric acid, which hydrolyze the proteins into smaller polypeptides. Pancreatic proteases (trypsin, chymotrypsin, and elastase), digestive hormones (secretin and cholecystokinin), and proteases (aminopeptidase N) further digest those polypeptides into tripeptides, dipeptides, and free amino acids in the small intestine. Dipeptides and tripeptides are absorbed by peptide transporter 1 (PepT1), a proton-dependent small oligopeptide transporter, which is located on the apical membranes of enterocytes, and they are digested into amino acids by cytoplasmic peptidases [64,65].

In addition to host-derived proteases, amino-acid-fermenting microbes also produce proteases to hydrolyze proteins and subsequently utilize the released amino acids for proteolytic fermentation or synthesis of microbial proteins (Figure 1). The abundant amino-acid-fermenting microbes detected in the human small intestine are mainly from the *Clostridium* cluster, the *Bacillus-Lactobacillus-Streptococcus* groups, and Proteobacteria [66]. Previous studies have suggested that these small intestinal microbes mainly utilize amino acids for the synthesis of bacterial proteins [66]. Amino acids are absorbed into enterocytes through various amino acid transporter systems including B^0^ depending on amino acid properties, transferred into the portal blood across the basolateral membrane, and then taken up into hepatocytes in the liver to be used for protein synthesis or gluconeogenesis [67,68]. For instance, glutamine participates in many key metabolic processes, such as protein synthesis, gluconeogenesis, inter-organ nitrogen transfer, nucleic acids biosynthesis, the immune response, and regulation of the cellular redox state [69].

### 3.2. Production of Dietary Metabolites in the Colon

#### 3.2.1. Carbohydrate Metabolites

Dietary carbohydrates are digested in the small intestine into glucose, galactose, and fructose and then absorbed by enterocytes. Sodium–glucose co-transporter (SGLT)1, glucose transporter (GLUT)5, and GLUT2 are responsible for the transport of glucose, galactose, and fructose across the brush border membrane or basolateral membrane [70]. A loss-of-function study using a gene knock out mouse model suggested that SGLT1 is important for fast glucose absorption; however, the presence of GLUT2 has not been shown to have any role in either apical glucose influx or incretin secretion [71]. When dietary fiber containing indigestible carbohydrates escapes the small intestine and reaches the colon, carbohydrate-targeting enzymes (e.g., glycoside hydrolases, polysaccharide lyases) released from anaerobic bacteria break down fiber into absorbable sugars. The released sugars are fermented by bacteria through metabolic pathways—the Embden–Meyerhof–Parnas pathway and the pentose-phosphate pathway—to build metabolic intermediates, resulting in the production of SCFAs [72]. SCFAs are representative metabolites that are synthesized de novo through microbial fermentation, mostly in the colon. The most abundant SCFAs (≥95% of total) in the gut are acetate (C2), propionate (C3), and butyrate (C4) [73]. Acetate, produced from pyruvate via acetyl-CoA or the Wood–Ljungdahl pathway, can be used as a substrate for butyrate production. Propionate is produced from lactate via the acrylate pathway or from succinate via the succinate pathway. Butyrate is produced from two molecules of acetyl-CoA via the phospho-transbutyrylase and butyrate kinase route or the butyryl-CoA:acetate CoA-transferase pathway [74]. Each enzymatic reaction involved in the production of each SCFA is regulated by specific microbes expressing the genes responsible for each biosynthesis pathway. Specifically, many enteric bacteria and Bacteriodetes produce acetate and propionate [39,75,76,77,78]. Firmicutes (*Lachnospiraceae* and *Ruminococcaceae*) are known to produce butyrate [75,76,77].

SCFAs are absorbed by cells like colonocytes or transported to tissues via simple diffusion or active transport using Na^+^-coupled or H^+^-coupled transporters such as SLC5A8 and SLC16A1 [79]. Some SCFAs (mostly acetate, but possibly some propionate) reach the circulation and can also directly affect metabolic responses in the peripheral tissues, including adipose, brain, and liver tissue [1,80].

#### 3.2.2. Lipid and Bile Acid Metabolites

Although, most bile acids and cholesterol are reabsorbed into the distal small intestine, some bile acids (approximately 5%) and cholesterol reach the colon and then dynamically interact with the gut microbiota [81]. The cholesterol evading reabsorption reaches the colon, at which time it is metabolized by the intestinal microbiota. Otherwise, it is excreted with feces [82]. The microbial activity on cholesterol is based on enzymatic reduction to produce coprostanone and coprostanol via two different pathways [83]. The first direct reduction of the 5–6 double bond by cholesterol reductase produces coprostanol. The second oxidation of the 3b-hydroxy group and isomerization of the double bond produce 4-cholesten-3-one by cholesterol oxidase (ChOx) or 3b-hydroxysteroid dehydrogenase/isomerase (HSD), respectively.

A small amount of escaped bile acids (<10%) from ileum re-absorption move into the colon, where bacteria transform primary bile acids to secondary and free bile acids. As shown in Figure 1, numerous secondary bile acids are produced by bacteria metabolism, including lithocholic acid (LCA) and deoxycholic acid (DCA). Firmicutes, including *Clostridium* cluster XVIa and XI, are known to produce secondary bile acids (i.e., LCA and DCA) from primary bile acids [84]. Furthermore, secondary bile acids undergo microbial isomerization, resulting in unique immunomodulatory properties. For example, the secondary bile acid DCA does not promote Treg, but its isomer, isoDCA promotes Treg differentiation [85]. Secondary bile acids result from the following bacterial activity through various reactions: deconjugation by bile salt hydrolases that hydrolyze amide bonds and 7-dehydroxylation resulting in transformation of the primary deconjugated bile acids into secondary bile acids [82]. While deconjugation reactions are mediated by various colonic bacteria, 7α-dehydroxylation is restricted to a limited number of intestinal bacteria, such as *Clostridium scindens* [81]. Bile salt hydrolase encoding genes have been found in gut microbes including *Bacteroides*, *Clostridium*, *Lactobacillus*, and *Bifidobacterium* [81]. The conversion of primary to secondary bile acids by 7a-dehydroxylases is probably one of the most physiologically relevant microbial transformations in humans [81]. Through 7a-dehydroxylation, primary cholic acid is transformed into secondary deoxycholic acid, and primary chenodeoxycholic acid is transformed into secondary lithocholic acid. 7a-dehydroxylation activities have been characterized in the genera *Eubacterium* and *Clostridium*, including in *Clostridium scindens* and *Clostridium hylemonae* species [81]. Intestinal microbes participate in the production of trimethylamine-*N*-oxide (TMAO), a well-known metabolite related to atherosclerosis development. Dietary sources including choline, phosphatidylcholine, L-carnitine, and other methylamine-containing nutrients provide substrates for microbiota-mediated production of trimethylamine (TMA). It has been suggested that TMA is absorbed into IECs and subsequently metabolized to TMAO by flavin monooxygenase in the liver [86,87,88].

Microbes in the colon synthesize sphingolipids, lipids with a sphingosine backbone de novo [89]. The synthesis of sphingolipids requires the serine palmitoyltransferase (SPT) enzyme and two precursors: the fatty acid palmitoyl coenzyme A (CoA) and the amino acid serine. Thus, the production of sphingolipids is restricted in SPT-expressing bacteria. Species in Bacteroidetes phyla such as Bacteroides fragilis are the major commensal bacteria that produce sphingolipids [90]. The metabolism of sphingolipids generates diverse metabolites such as ceramide, ceramide-1-phophate, glucosylceramide, sphingomyelin, sphingosine, and sphingosine-1-phosphate [91].

#### 3.2.3. Protein Metabolites

Although most protein digestion and absorption occur in the small intestine, some undigested proteins and amino acids move into the large intestine together with nitrogenous compounds. The transferred proteins are mostly degraded in the descending colon because of the luminal pH difference (slightly acidic in ascending to neutral in descending) and activity of proteases produced by colonocytes and microbes [92]. Moreover, large intestinal microbes preferentially involve a carbohydrate metabolism rather than protein metabolism. Thus, protein fermentation mainly occurs in the descending colon where carbohydrate depletion has already occurred [92]. In the large intestine, amino-acid-fermenting microbes utilize amino acids intensively for proteolytic fermentation or de novo synthesis of amino acids, rather than the synthesis of microbial cell components [93,94]. The predominant amino-acid-fermenting microbes detected in the human large intestine are *Bacteroides* and *Propionibacterium.* The abundance of *Bacteroides* is highly correlated with the consumption of a high protein diet including various types of meat [95]. In addition, other proteolytic microbes, such as *Streptococci*, *Clostridium*, *Baccilus*, and *Staphylococcus*, are detected in the colon [92,96]. The microbes generate diverse metabolites, including branched-chain fatty acids (BCFAs), SCFAs, essential amino acids, polyamines, and ammonia through the proteolytic fermentation of amino acid precursors.

Amino acids serve as precursors to SCFAs during proteolytic fermentation, similar to carbohydrate fermentation. Acetate is generated from the catabolism of glycine, alanine, threonine, glutamate, lysine, and aspartate. Butyrate is produced from the catabolism of glutamate, lysine, and propionate from alanine and threonine [97]. Approximately 5–10% of SCFAs are BCFAs, such as isobutyrate, isovalerate, and valerate [98]. BCFAs are produced during the fermentation of branched chain amino acids: leucine, valine, and isoleucine.

Tryptophan is metabolized by kynurenine, serotonin, and indole pathways. Approximately 95% of tryptophan is metabolized through the kynurenine pathway, which involves a cascade of enzymatic reactions [99]. Indoleamine 2,3-dioxygenase (IDO), produced by immune cells and IECs, initiates the degradation of tryptophan and leads to the production of metabolites including kynurenine, kynurenic acid, 3-hydroxykynurenine (3-OHKyn), 3-hydroxyanthranilic acids (3HAA), and quinolinic acid. A minor portion of tryptophan (~1%) is converted into serotonin by the enzyme tryptophan hydroxylase, which is expressed by enterochromaffin cells. Both kynurenine and serotonin production pathways are regulated by host cells. On the other hand, approximately 5% of tryptophan is catabolized by gut microbes (e.g., *Lactobacilli* and *Clostridium sporogenes*), which produce tryptophanase and decarboxylase, resulting in the generation of tryptamine and indole metabolites including indole-3-propionic acid, indole-3-acetic acid, and indole-3-aldehyde [100].

## 4. Mucosal Immune Regulatory Mechanism of Gut Metabolites

The balance of the intestinal immune system is controlled by various extrinsic factors. As intestinal tissues are constantly exposed to trillions of gut microbes and dietary components, not surprisingly, they are one of the most important regulatory factors for intestinal immunity. A number of studies have reported that, together with dietary factors, an altered gut microbiome is closely associated with gut inflammatory diseases such as IBD. While underlying mechanisms are yet to be fully understood, abnormal intestinal immune responses induced by altered gut metabolite profiles are considered to be a critical reason for the development of diseases in the gut. As a number of studies have unveiled mechanisms for explaining the role of the diet–microbiota–metabolite axis in the regulation of host immunity, we summarize the emerging molecular and cellular mechanisms involved in intestinal immunity regulation by major gut metabolites from carbohydrates, lipids, bile acids, and amino acids.

### 4.1. Carbohydrate Metabolites

Lactic acid bacteria, the most popular probiotic strain, have been used to enhance health for a long time. One of the mechanisms suggested to be responsible for the positive effects of lactic acid bacteria is lactate production. Lactate is a major component of lactic acid bacteria-fermented food and can regulate critical functions of macrophages, DCs, T cells, and IECs. Lactate is known to have immunomodulatory effects in inflammatory environments [101]. GPR81, a cell-surface receptor for lactate, regulates intestinal homeostasis and protects mice from experimental colitis. A study found that GPR81-deficient mice display imbalanced CD4^+^ T cell subsets, characterized by increased Th1/Th17 and decreased Treg. GPR81 signaling in colonic DCs and macrophages is important for suppressing inflammation and restoring colonic homeostasis [102]. Lactate produced in the gut is recognized by GPR81 expressed on Paneth cells and stromal cells and activates Wnt/β-catenin signaling for gut homeostasis [8]. Microbiota-derived lactate has been suggested as a major factor in the induction of enterocyte hyperproliferation in starvation-refed mice [103]. Furthermore, lactate and pyruvate produced by bacteria induce dendrite protrusion of intestinal CX3CR1^+^ macrophages in a GPR31-dependant manner, enhancing local immune responses and providing a high level of resistance against intestinal *Salmonella* infection [104].

**Figure 2:**
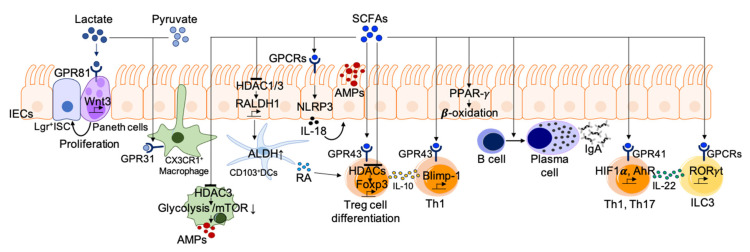
Immune regulation by carbohydrate metabolites. Lactate and pyruvate induce dendrite protrusion of intestinal CX3CR1^+^ macrophages via GRP31 to enhance the immune response. Lactate also maintains gut homeostasis via Wnt/β-catenin signaling activation to induce intestinal stem cell proliferation. SCFAs act as HDAC inhibitors or stimulators for GPCRs to regulate intestinal immune homeostasis. SCFAs induce Treg cell differentiation, plasma B cell differentiation, and IL-22 production from ILC3 as well as Th1 and Th17 cells. Additionally, SCFAs regulate the activity of certain macrophages and dendritic cells (DCs). AhR, aryl hydrocarbon receptor; ALDH, aldehyde dehydrogenase; AMPs, anti-microbial peptides; GPCRs, G protein coupled receptor; HDAC, histone deacetylase; HIF1α, hypoxia-inducible factor 1-alpha; ISC, intestinal stem cell; PPAR-γ, peroxisome proliferator-activated receptor-γ; RA, retinoic acid.

SCFAs, which are well-known microbial metabolites, have been investigated extensively to determine their role in the maintenance of immune homeostasis through the regulation of epithelial integrity as well as innate and adaptive immunity (Figure 2). The involvement of SCFAs in the regulation of a variety of intestinal immune cells occurs mainly through three distinct mechanisms: (1) the integration of cellular metabolism, (2) the inhibition of histone deacetylases (HDAC), and (3) G-protein coupled receptor (GPCR) activation.

(1) Integration of cellular metabolism. As SCFAs are small, they can be absorbed into various cells and are then integrated into cellular metabolism. The most prominent role of SCFAs is as an energy source for colonocytes and immune cells as well [73]. It is well known that dietary fiber and SCFAs support intestinal epithelial proliferation. SCFAs are converted to acetyl-CoA for energy production through the tricarboxylic acid cycle and lipid synthesis [73]. In particular, butylate (C4), known as the primary energy source for colonocytes, activates peroxisome-proliferator-activated receptor (PPAR)-γ signaling, which maintains the energy metabolism of colonocytes toward the mitochondrial β-oxidation of fatty acids [105,106]. The PPAR-γ dependent activation of mitochondrial β-oxidation preserves epithelial hypoxia and limits host-derived nitrate availability in the lumen, thereby preventing the dysbiotic expansion of pathogenic facultative anaerobic bacteria [106]. Immune cells need a significant portion of metabolic building blocks during activation. For instance, adaptive lymphocytes utilize available nutrients to produce energy (e.g., adenosine triphosphate, ATP), undergo biogenesis of cellular components, and produce effector molecules such as antibodies and cytokines. Moreover, SCFAs integrate cellular metabolism to support functional changes in adaptive immune cells. Both glycolysis and oxidative phosphorylation for ATP production are prerequisite steps in B cell activation and plasma B cell differentiation [107]. Kim and colleagues reported that dietary fiber and SCFAs support plasma B cell differentiation through metabolic integration [35]. In fact, SCFA (C3 or SCFA mixture) administration was shown to rescue impaired germinal center formation and IgA expression in mice fed a low-fiber diet. SCFAs promote metabolic processes such as fatty acid oxidation and mitochondrial respiration in B cells during their activation. These results suggest that SCFAs partially mediate the positive effects of dietary fiber intake on gut IgA responses by supporting metabolic processes [35].
(2) Act as HDAC inhibitors. Histone acetylation is an epigenetic modification method for histone, which promotes the formation of open chromatin by adding acetyl groups to the lysine residues and activates transcription. On the contrary, HDACs remove acetyl groups and repress gene transcription. SCFAs inhibit HDACs, thereby promoting gene expression in epithelial cells, T cells, and macrophages. SCFAs promote gut barrier integrity through the induction of AMPs such as RegIIIγ, and β-defensin in epithelial cells via the activation of mammalian target of rapamycin signaling pathway (mTOR) and STAT3 [108,109]. Such functional regulation is mediated by HDAC inhibition together with AMP-activated protein kinase (AMPK) activation. Diet-derived SCFAs are also known to stimulate IECs to induce mucosal tolerogenic DCs through HDAC inhibitory action [110]. SCFAs induce an increase in RALDH1 by inhibiting HDAC1 and HDAC3 in IECs. The increased RALDH1 expression in IECs correlates with the ALDH activity of CD103^+^ tolerogenic DCs, along with increased numbers of intestinal Treg and high luminal IgA [110]. Among SCFA species, butyrate has strong HDAC-inhibitory activity. HDCA3 inhibition by butyrate induces the differentiation of macrophages possessing antimicrobial activity. The inhibition of metabolic programming in macrophages by glycolysis and mTOR inhibition results in the enhancement of AMPs, such as S100A8/A9/A12 and lysozymes [111]. SCFAs, as HDAC inhibitors, also affect T cell differentiation through the regulation of metabolic sensors [112]. For instance, HDAC inhibition by SCFAs enhances the acetylation of key metabolic sensors for the mTOR pathway (p70S6 kinase and phosphorylation rS6), supporting IL-10 expressing Treg differentiation. Butyrate directly induces Treg generation to provide immune tolerance in response to commensal bacteria by enhancing acetylation of the genetic locus for *Foxp3* [34,113,114]. In addition to Treg, SCFAs can directly promote naïve CD4^+^ T-cell differentiation into Th1 or Th17 cells [112].
(3) GPCR activation. SCFAs can activate many cell types via GPCRs expressed on diverse cells including IECs, neutrophils, macrophages, DCs, B and T cells, and ILCs. Acetate is known to be a ligand of GPR43, and propionate is a ligand of GPR43 and GPR41. Butyrate induces activation through GPR41 and GPR109A [115]. SCFAs facilitate the production of inflammatory effector molecules by IECs in response to immune challenges including ethanol-induced breach, TNBS, and *Citrobacter rodentium* infection [116]. GPR41- and GPR43-deficient animals display abnormally low inflammatory responses in the gut [116]. SCFAs recognized by GPR43 and GRP109A on IECs activate the NLRP3 inflammasome, leading to IL-18 secretion for maintenance of the gut barrier integrity in chronic inflammation and colorectal cancer cases [115,117,118,119]. SCFAs activate antigen-specific Th2 cells to produce the immunosuppressive cytokines IL-10 [120] and IL-22 [121,122] via GPCRs. The binding of butyrate to GPR41 promotes IL-22 production in Th1 and Th17 as well as ILC3 by upregulation of hypoxia-inducible factor 1α and AhR [122]. The binding of propionate to GPR43 regulates colonic IL-22 expression in ILC3 via AKT and STAT3 signaling. GPR43-deficient ILC3s enhance the susceptibility to colonic inflammation and *C. rodentium* infection [121]. Neutrophils express the SCFA receptor GPR43, and its activation induces chemotaxis and functional activation, which could play roles in intestinal homeostasis. SCFAs also indirectly regulate Treg generation by inducing the expression of anti-inflammatory molecules (IL-10 and Aldh1a) on DCs and macrophages [115]. Propionate also induces an increase in GPR15 expression, which regulates the homing of Treg to the large intestine [34,123].

### 4.2. Lipid and Bile Acid Metabolites

**Figure 3:**
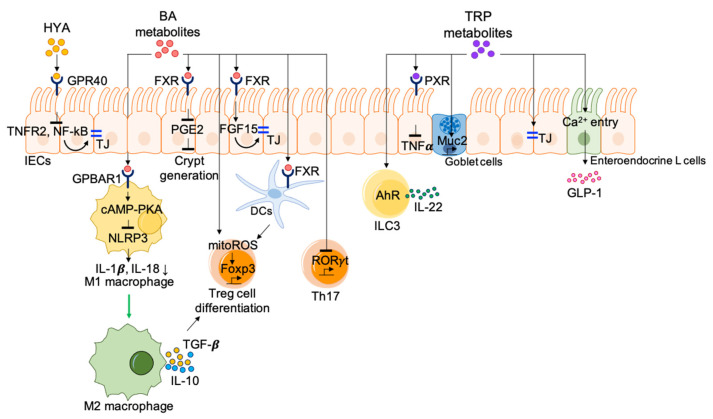
Immune regulation by metabolites derived from lipids and amino acids. Microbial metabolites produced from lipids and amino acids regulate intestinal homeostasis. HYAs from lipids and tryptophan metabolites induce the expression of TJ proteins on epithelial cells to fortify intestinal barrier integrity. Bile acid metabolites inhibit the differentiation of Th17 cells but promote Treg cell differentiation through the production of mitoROS and induce the conversion of M1 macrophages to M2 macrophages. Also, bile acid metabolites help to maintain gut barrier integrity through accelerating crypt regeneration. Tryptophan metabolites enhance intestinal homeostasis through AhR activation to promote IL-22 production in ILC3 and stimulation of enteroendocrine L cells to produce GLP-1. BA, bile acids; EFG15, epidermal growth factor 15; FoxP3, forkhead box P3; FXR, farnesoid X receptor; GLP-1, glucagon-like peptide-1; GPBAR1, G protein-coupled bile acid receptor 1; HYA, 10-hydroxy-cis-12-octadeccenoid acid; IECs, intestinal epithelial cells; mitoROS, mitochondrial reactive oxygen species; MUC2, mucin 2; PGE2, prostaglandin E2; PXR, pregnane X receptor; RORγt, RAR-related orphan receptor gamma; TJ, tight junction; TNFα, tumor necrosis factor alpha; TRP, tryptophan.

A variety of microbial metabolites produced from lipids have significant impacts on the immune system through the regulation of functional activities of immune cells (Figure 3). The PUFAs ω-3 and ω-6 are essential fatty acids that are metabolized into bioactive lipid mediators through reactions that are mediated by several oxidative enzymes. It is thought that ω-3 PUFAs dampen inflammatory reactions, whereas ω-6 PUFAs have pro-inflammatory properties [124]. It has been reported that the resolution of inflammation by ω-3 PUFAs plays a role in the recovery of intestinal immune homeostasis via GPR120 [125]. In addition to GPCRs, metabolites derived from ω-3 PUFAs activate a lipid-sensing nuclear receptor PPAR-γ for the maintenance of intestinal homeostasis [126]. The resolution process includes the termination of neutrophil recruitment, the suppression of pro-inflammatory responses, the stimulation of cell debris clearance by macrophages, and tissue remodeling. As mentioned previously, commensal bacteria metabolize PUFAs to produce microbial metabolites such as CLA, HYA, oxo fatty acids, and hydroxy fatty acids. The administration of CLA protects against dextran sodium sulfate (DSS)-induced colitis as well as CD4^+^CD45RB^hi^ T cell-induced colitis through a PPAR-γ-dependent mechanism. PPAR-γ activation by CLA suppresses the activity of nuclear factor-kB (NF-κB) acting as a central mediator of inflammation and regulates colon epithelial cell maturation related gene expressions [127]. PPAR-γ is essential for maintenance of innate antimicrobial immunity regulating expression of β-defensin in the colon [128]. The administration of HYA ameliorates experimental colitis by enhancing the expression of tight junction (TJ) proteins on epithelial cells. TJs constitute a barrier to the passage of ions and molecules through the paracellular pathway and to the movement of molecules including, but not limited to, proteins and lipids between the apical and basolateral sides of the plasma membrane. TJ proteins are multiprotein junctional complexes that are observed at the tight junctions of epithelial, endothelial, and myelinated cells. Occludin and claudins are integral TJ proteins that are capable of interacting adhesively with complementary molecules on adjacent cells, working together with another class of TJ protein, zonula occludens, that is required for the coordination of signals coming from the plasma membrane. Barrier integrity regulation by HYA is initiated through the interaction with GPR40, which suppresses TNFR2 expression via the NF-κB and MEK-ERK pathways [129].

Host enzymatic reactions produce primary bile acids, and gut microbiota further produce various secondary bile acids that work as signaling molecules via interactions with receptors, such as the farnesoid X receptor (FXR), the vitamin D receptor, G protein-coupled bile acid receptor 1 (GPBAR1, also known as TGR5, GPCR19, or M-BAR), and the pregnane X receptor (PXR) [130,131,132,133]. Ablation of GPBAR1, FXR, or PXR causes increased susceptibility to chemically induced intestinal inflammation [134,135,136,137]. Specifically, bile acids activate GPBAR1 or FXR in macrophages, resulting in the differentiation of anti-inflammatory macrophages and the reduction in pro-inflammatory cytokine production via the inhibition of NLRP3-dependent inflammasomes and NF-κB-dependent signaling pathways [138]. Exposure of the GPBAR1 agonist shifts inflammatory M1 to anti-inflammatory M2 macrophages, consequently increasing Treg via the expression of IL-10 and TGF-β [136,139]. In terms of Treg differentiation, multiple groups have reported that bile acids play a significant role in the induction of intestinal Treg via the activation of bile acid receptors. IsoDCA, a secondary bile acid generated via the microbial epimerization of cholic acid, promotes Treg differentiation through FXR signaling on DCs [85]. Interestingly, in this study, researchers utilized a synthetic biology approach and designed minimal microbial consortia containing IsoDCA-producing bacteria to promote the RORγt^+^ Treg pool in the gut [85]. Intriguingly, the homeostasis of RORγt-expressing Treg is regulated by the gut bile acid pool rather than by a single type of primary or secondary bile acid [140]. A mixture of certain primary bile acids (e.g., cholic acid/ursodeoxycholic acid mix, chenodeoxycholic acid/ursodeoxycholic acid mix, cholic acid/chenodeoxycholic acid/ursodeoxycholic acid mix) and secondary bile acids generated from bacterial oxidation and dihydroxylation pathway preferentially maintains colonic frequencies of RORγt^+^ Treg and Foxp3^+^ Treg. This shows the importance of the host–microorganism biliary network in the maintenance of immune homeostasis in the gut [140]. In addition to their effect on Treg differentiation, bile acid metabolites are able to directly regulate the balance between Th17 and Treg cells. 3-oxoLCA signaling through RORγt inhibits the differentiation of Th17 cells. Another bile acid metabolite, isoalloLCA, also increases Treg differentiation through the enhancement of mitochondrial reactive oxygen species (mitoROS)-dependent Foxp3 expression [141]. One important regulatory function of secondary bile acids is the fortification of gut barrier function through multiple mechanisms, including the maintenance of intestinal barrier integrity and the inhibition of pathogen colonization. DCA downregulates prostaglandin E2 synthesis in an FXR-dependent manner, thereby accelerating intestinal crypt regeneration and wound repair [142]. Administration of a mixture of LCA and ursodeoxycholic acid helps to maintain gut barrier integrity through the activation of the FXR–FGF15 pathway [143].

Lipids synthesized by commensal bacteria influence intestinal immune homeostasis via negative regulation of iNKT proliferation during neonatal development [89]. iNKT cells respond quickly upon CD1d protein-presented lipid antigen recognition and secrete high levels of inflammatory cytokines (IL-13 and IFN-γ), resulting in epithelial destruction and severe inflammation. A commensal sphingolipid produced by *B. fragilis*, α-galactosylceramide (α-GalCer), limits the expansion of colonic iNKT during early postnatal life via interactions with the atypical MHC class I molecule CD1d. Thus, it prevents an excessive inflammatory response during colitis progression [89].

### 4.3. Protein Metabolites

The absorbed amino acids are important for the maintenance of IEC integrity (Figure 3). The protective functions of amino acids in the intestine are closely associated to the apoptosis and proliferation of IECs, the expression of TJ proteins, the alleviation of intestinal inflammation and oxidative stress by inhibiting NF-κB signaling pathway, and the activation of the nuclear erythroid-related factor 2 (Nrf2) signaling pathway. The catabolism of glutamate, glutamine, and aspartate provides ATP required for metabolic processes of IECs [144]. L-glutamine enhances intestinal enterocyte growth in porcine epithelial cells, IPEC-1, cultured in glutamine-free media. L-glutamine treatment activates mTOR independently of AMPK [145]. In addition, some amino acids are essential for limiting cellular stress on epithelial cells. For instance, glutathione synthesized from glutamate, glycine, and cysteine is a powerful antioxidant that protects IECs from oxidative damage [146]. Furthermore, amino acids act as essential precursors for the synthesis of important proteins and peptides such as mucins, immunoglobulins, and defensins for maintaining normal gut structure and function.

Among amino acid metabolism processes related to gut physiology, tryptophan metabolism is the most widely reported, as tryptophan metabolites play an important role in the regulation of intestinal immune homeostasis. A lack of tryptophan or its amino acid transporter, B^0^AT1 (SLC6A19) in the diet decreases the expression of anti-microbial peptides in enterocytes, altering the gut microbiome, which increases susceptibility to intestinal inflammation [147]. Metabolites derived from tryptophan catabolism regulated by the host or microbiota act as aryl hydrocarbon receptor ligands and control the transcription of a wide variety of target genes, such as IL-22 and IL-10 [148,149,150]. Indole metabolites derived from microbial tryptophan metabolism such as indole 3-aldehyde and indole 3-propionic acid activate AhR to promote IL-22 production in ILC3, which increases the expression of anti-microbial peptides and consequently enhances intestinal homeostasis [148]. A deficiency of caspase recruitment domain 9 (CARD9), an IBD susceptibility gene, alters the gut microbiota, causing a failure to produce AhR ligands because of impaired tryptophan metabolism. This leads to a reduction in IL-22 and, thus, greater susceptibility to colitis [151]. Indole 3-propionic acid, a symbiotic bacterial indole metabolite, activates PXR on IECs, which regulates TLR4-mediated control of the intestinal barrier function [152]. Oral administration of indole 3-propionic acid ameliorates DSS-induced colitis with increased colonic epithelial IL-10R1 expression [153]. Indole acrylic acid produced by commensal *Peptostreptococcus* species has anti-inflammatory effects, resulting in enhanced IL-10 production and increased Muc2 expression [154]. Indole promotes intestinal epithelial barrier function by inducing the expression of tight-junction-associated genes such as *Cldn7*, *Ocln*, and *Tjp1* [154]. Even brief exposure of indole enhances the entry of Ca^2+^ into enteroendocrine L cells, leading to glucagon-like peptide-1 production [155]. In addition to the indole pathway, the kynurenine pathway participates in the regulation of immune homeostasis. Considering that IDO1 initiates the kynurenine pathway, IDO1 activity and its regulatory function in association with kynurenine pathway have been well studied. The activation of IDO1 in DCs and macrophages results in increased production of kynurenine, which induces Treg generation by activating AhR and ultimately promotes homeostasis [156,157,158]. Kynurenine generated by IFNγ-stimulated IDO1 induction increases IL-10R1 expression on intestinal epithelia cells, resulting in the mitigation of colitis [159]. Furthermore, overexpression of IDO1 in the intestinal epithelium augments resistance to colitis via the promotion of secretory cell differentiation and mucus production [160].

## 5. Summary

Intestinal homeostasis requires stringent regulation of immune responses. Multiple types of innate and adaptive immune cells maintain the balance between tolerance and activation in intestinal tissues facing potential antigens, including dietary materials, commensal bacteria, and sometimes unexpected pathogens. The signals derived from antigenic materials in the lumen of intestine act as key regulatory factors to regulate intestinal inflammation.

Through both host and microbial enzymatic activities, dietary components can be converted into various gut metabolites with unique functional activities. Following recent progress in the field of nutritional immunology, we are starting to understand the molecular and cellular mechanisms through which gut metabolites regulate intestinal immune cells. Unveiled mechanisms include the activation of specific cell surface receptors, the control of gene expression by epigenetic regulation, and the integration of cellular metabolism. These mechanistic studies help to develop new strategies to control intestinal diseases such as IBD and colorectal cancer.

To develop effective strategies, we need to focus on the following issues raised from recent progress in metabolite studies: (1) firstly, metabolites have different effects on intestinal inflammation. Several contradicting reports have suggested that metabolites impact intestinal inflammatory reactions differently. For example, SCFAs have a dual role in T cell differentiation and intestinal inflammation. The explanation for this issue is probably that the role of gut metabolites in the regulation of intestinal inflammation can switch based on the occurrence of an inflammatory disease or cancer vs. under homeostatic conditions. Thus, we need to put more effort into understanding how gut metabolites differentially affect immune cell functions in the context of intestinal immunity. (2) The second issue is the balance of metabolites in the gastrointestinal tract. Intestinal immune cells are exposed to the diverse gut metabolite pool. Thus, changes in intestinal immunity can be explained by actions of multiple metabolites not just a single type of metabolite. We need to have an integrated approach to understand complex metabolite signals. For example, we may need to determine whether a certain metabolite affects the functional activities of another metabolite on intestinal immunity. (3) The third strategy is to manipulate the diet–metabolite–microbiota axis. To induce the production of particular metabolites for the control of intestinal immunity, it could be necessary to directly alter the diet or intervene with microbiota communities. A practical strategy to control the intestinal environment based on the physiological and immunological conditions required in the gastrointestinal tract needs to be developed. Collectively, further integrative mechanistic studies for gut metabolites will enhance the development of effective dietary strategies to prevent or cure intestinal diseases.

## Data Availability

Not applicable.
